# University students’ strategies of coping with stress during the coronavirus pandemic: Data from Poland

**DOI:** 10.1371/journal.pone.0255041

**Published:** 2021-07-26

**Authors:** Anna Babicka-Wirkus, Lukasz Wirkus, Krzysztof Stasiak, Paweł Kozłowski

**Affiliations:** 1 Institute of Pedagogy, Pomeranian University in Słupsk, Słupsk, Poland; 2 Institute of Pedagogy, Faculty of Social Science, University of Gdańsk, Gdańsk, Poland; 3 Department of Material Criminal Law and Criminology, Faculty of Law and Administration, University of Gdańsk, Gdańsk, Poland; Shahjalal University of Science and Technology, BANGLADESH

## Abstract

The COVID-19 pandemic has changed the functioning of universities worldwide. In Poland, the transfer to online teaching was announced without prior warning, which radically changed students’ daily functioning. This situation clearly showed the students’ helplessness and difficulties with coping with this new, stressful situation, highlighted in many previous studies. A sudden and far-reaching change in daily functioning caused anxiety, depression, and stress in this group. Thus, from a pedagogical and psychological point of view, it is pertinent to examine the students’ strategies of coping with stress caused by the COVID-19 pandemic. To this end, in 2020, a sample of Polish students was anonymously measured using the Mini-COPE questionnaire. Data was gathered from 577 students from 17 universities. The statistical analysis showed that during the coronavirus pandemic, Polish students most often used the coping strategies of: acceptance, planning, and seeking emotional support. Such factors as age, gender, and place of residence influenced the choice of specific strategies of coping with stress during the COVID-19 pandemic. The results also showed that the youngest students had the lowest coping skills. The results allow for concluding that the students’ maladaptive strategies of coping with stress, especially during the pandemic, may result in long-term consequences for their psychophysiological health and academic achievements. Thus, based on the current results and on the participatory model of intervention, a support program for students is proposed which would involve psychological, organizational, and instrumental support.

## Introduction

University studies are a stressful period as they mean the transition to independent, adult life. Beginning studies can be stressful to many students, since it means the necessity to establish new relationships, develop new studying habits related to the chosen program, cope with overwork, learn time management, and often also change one’s place of residence [1, 2]. In its later stages, university education is related to new, further stressors, such as concern over being able to find employment after graduation. Studies thus far show that many students struggle to cope with these stressors and that the incidence of stress among students is increasing [3, 4]. Among other consequences, it has a negative impact on mental health [2, 5]. In the US, around 10% of university students reported suffering from depression [2], and this proportion has increased to 15% since 2000 [6–9]. A significant causal factor behind this increase is the stress related to studying.

Stress is undoubtedly a part of students’ lives and it may impact their ways of coping with the demands of university life. Their daily responsibilities involve numerous challenges which lead to stress [10]. Results from various studies carried out thus far show a clear increase in mental health problems among students [11]. As some of them indicate, there is also an urgent need to assess the impact of the current pandemic on students’ mental health and wellbeing [12], which legitimizes carrying out such studies in various countries, including Poland.

In 2020, a new situation appeared which necessitates a different approach to stress and its causal factors–the SARS-CoV-2 virus. Data published by the Johns Hopkins University indicates that thus far, over 100 million people have become infected with COVID-19, and around 2.5 million have died [13]. The COVID-19 disease affects everyone, including students [14, 15], since even those who have not been infected are subject to various restrictions which many countries have implemented to limit the spread of the disease. The reality of the pandemic has also negatively impacted the students’ quality of social life. Studying at a university is also a period of establishing new relationships and intense social life. This is facilitated by the fact that young people exhibit greater levels of extraversion and openness to experience than do older people [16]. Studies show that contacts with others positively influence quality of life [17]. Lack of regular contact with friends throughout all phases of the coronavirus pandemic, results in loneliness, which might not be fully mitigated by regular contacts via telephone or other means [18]. These conclusions are supported by evidence from studies carried out in Great Britain (with participants aged between 13 and 25 years), in which young people reported having lost support, daily routine, social ties, and experiencing anxiety, loneliness, and loss of motivation and aim. Higher incidence of depression and anxiety, both during as well as after periods of social isolation, was also confirmed [19, 20]. This may lead to harmful social and psychological consequences [21–23].

In response to the pandemic, most countries have implemented severe restrictions in societal functioning which comprise many spheres of life: social, economic, cultural, and educational. They led to limited interpersonal contacts, changes in the mode of education (online teaching), and reduced economic activity. As a result, an economic recession has affected nearly all countries (including Poland) [24], which worsened the material conditions of many people (increased unemployment). This significantly impacts students, as it intensifies their concerns about being able to find or retain a job and thus support themselves during their studies and after graduation. Essen and Owusu showed that work and studies are the most frequent causes of stress for students [25]. Historical data shows that previous pandemics have negatively impacted young people’s material conditions, which had long-term consequences for their physical and mental health as well as academic achievement [26]. For many students, COVID-19 has additionally complicated their current plans and changed their mode of functioning.

More recently, Matthew H. E. M. Browning et al. identified a range of psychological consequences of the COVID-19 pandemic on students’ psychosocial functioning. All students in the sample indicated that the pandemic impacted them negatively, with 59% reporting a high level of psychological impact [27]. Other studies on the effects of the pandemic on student mental health also show greater stress, anxiety, depression symptoms, concerns for own and one’s family’s health, reduced social interactions, and increased concerns over academic achievements. Students try to cope with stress, seek support from others, and prefer either negative or positive coping strategies [11, 28].

The COVID-19 situation, its rapid spread, insufficient preparation, and significant changes in everyday functioning, including university culture, may contribute to increased stress among students. When not managed properly, chronic stress leads to emotional and psychosomatic consequences which manifest through physical, cognitive, and emotional exhaustion as well as depersonalization and lowered professional–in case of students, academic–efficiency [29]. The consequences of stress lower efficiency, productivity, and engagement in life activities as well as the satisfaction with their results [30, 31]. As Adler and Park point out, effective coping with stress might buffer the impact of stressful events on the physical and mental health, and individuals differ with regards to the coping strategies they use [32]. Therefore, the aims of the study were: identifying the students’ dominant strategies of coping with stress in the pandemic situation, assessing the influence of sociodemographic factors on the dominant coping strategies, and diagnosing differences in the students’ coping strategies depending on expected social support and its sources.

The stress and coping concept is the most popular study approach, also explaining the mechanisms mediating between personality and disease. Currently, the transactional model of stress by Lazarus and Folkman [33] is employed increasingly frequently. It posits mutual interactions between people and their environment. This model served as the theoretical basis of the current study. The perception of stress is a subjective and variable phenomenon. Particular attention is paid to the processes of coping with stress, which decide the positive and negative impact of stress on the individual. Using different strategies of coping with stress involves mobilizing cognitive and behavioral resources to meet the demands which are subjectively perceived as surpassing personal capabilities. The course of the coping process depends on personal resources and social support. It can also lead to various behaviors which have negative health effects (substance use) or are maladaptive [34]. Also, according to Lazarus and Folkman, coping with stress might be related to negative health behaviors [35]. Metzger et al. analyzed the frequency of negative health behaviors among students. They found that increased alcohol consumption and risky sexual behaviors are typical for people at risk for significant stress [36]. Styles of coping with stress are determined by gender, education, age, health, well-being, the nature of the stressful situation, personality factors, and others [20]. Efficient use of emotions allows for more effective problem solving, while venting anger and frustration and denial of reality are potentially destructive reactions to stress [37]. Expressing emotions might also lead to lower depression and hostility levels in stressful situations [38]. Some authors distinguish between emotion-focused and problem-focused coping styles, while others distinguish active and avoidant coping or identify maladaptive coping strategies (denial, substance use, venting of negative emotions) which allow for lowering subjectively experienced stress [39–41].

## Methods

### Research questions

The study concerned students’ strategies of coping with stress during the pandemic. The following research questions were put forward:

1. What strategies of coping with stress are most often used by students during the coronavirus pandemic?
2. What is the relationship between sociodemographic variables and the dominant coping strategies among students?
3. How does anticipated support differentiate the coping strategies used by students?

### Study population and procedure

In 2019, 1.230 million students studied at around 400 universities in Poland. Sixty-five percent were full-time students. Seventy-three percent studied at public (national) universities. The number of foreign students is relatively low in Poland, being only 61 thousand in 2019. Moreover, a decisive majority—around 60%—of students in Poland are women [42, 43]. This proportion reaches 65% for MA studies. Meanwhile, in the EU in general, women comprise around 54% of students [44].

In early spring of 2020, soon after online teaching was instituted, the questionnaire was distributed to students of four randomly chosen Polish universities. Those students who filled out the online questionnaire were also asked to share it with their acquaintances from other universities. Using snowball sampling method was determined by difficulties in reaching students directly, as well as by their reluctance, especially in the first phase of the pandemic in Poland, to take part in studies and fill out online questionnaires. Having students to invite their acquaintances to also take part in the study allowed for gathering a relatively large sample in a short time. There were no missing data, since the online platform did not allow for submitting incomplete questionnaires.

Participation in the study was voluntary. Informed written consent was obtained from every participant. Before participants started to fill out the online study questionnaire, they had to read the information about the project and its aims and checked the option ’I agree to take part in the study’. The data were analyzed anonymously. The research project and its procedure were approved by the Commission of Bioethics and Human Rights.

Using the snowball sampling method, data from 17 Polish institutions was obtained: universities, technical universities, medical universities, and higher vocational schools. This allowed for measuring coping strategies during the pandemic among students from various universities in various regions of Poland. However, it has to be noted that snowball sampling does not allow for generalizing the results to the entire student population in Poland. Nevertheless, based on the obtained data, certain trends in coping strategies among social sciences students can be shown.

The study took place in April-May 2020. Five hundred and seventy-seven complete questionnaires were collected. Table 1 shows the demographic characteristics of the sample divided by universities.

**Table 1:** Sociodemographic characteristics of the sample.

		UG	AP	UAM	UW	UJK	DU	Total
**Gender**	Female	89.5%	91.2%	87.5%	86.9%	97.5%	86.5%	89.6%
	Male	10.5%	8.8%	12.5%	13.1%	2.5%	13.5%	10.4%
**Age**	18–20	12.5%	41.2%	26.6%	38.4%	26.6%	29.7%	24.8%
	21–24	60.5%	58.8%	56.3%	57.6%	59.5%	70.3%	59.5%
	25–30	17.5%	0.0%	7.8%	4.0%	8.9%	0.0%	9.7%
	31 and over	9.5%	0.0%	9.4%	0.0%	5.1%	0.0%	6.1%
**Place of residence**	Village	21.5%	61.8%	43.8%	24.2%	55.7%	37.8%	35%
	Town up to 20 thousand residents	6.5%	14.7%	13.3%	5.1%	6.3%	13.5%	8.7%
	Town with between 20 and 100 thousand residents	22.5%	14.7%	28.1%	15.2%	6.3%	18.9%	19.6%
	City with over 100 thousand residents	49.5%	8.8%	14.8%	55.6%	31.6%	29.7%	36.7%
**Year of study**	I Undergraduate	14.0%	44.1%	32.0%	31.3%	20.3%	29.7%	24.6%
	II Undergraduate	6.5%	50.0%	23.4%	28.3%	6.3%	18.9%	17.3%
	III Undergraduate	20.0%	0.0%	20.3%	24.2%	8.9%	18.9%	18.0%
	I Graduate	22.5%	0.0%	7.0%	5.1%	5.1%	8.1%	11.4%
	II Graduate	23.0%	0.0%	7.0%	5.1%	12.7%	0.0%	12.1%
	I-III uniform Master’s studies	2.5%	5.9%	10.2%	4.0%	39.2%	24.3%	11.1%
	IV-V uniform Master’s studies	11.5%	0.0%	0.0%	2.0%	7.6%	0.0%	5.4%
**Mode of study**	Full-time	60.5%	100%	82.0%	97.0%	74.7%	94.6%	78.0%
	Extramural	39.5%	0.0%	18.0%	3.0%	25.3%	5.4%	22.0%

The data was divided into six groups based on the number of students from each given university who took part in the study. The largest group were the students from the University of Gdańsk (UG). Next, the sample comprised students from: Adam Mickiewicz University in Poznań (UAM) - 22.2%, University of Warsaw (UW) - 17.2%, Jan Kochanowski University of Kielce (UJK- 13.7%, and Pomeranian University in Słupsk (5.9%). Due to a low number of students from other universities, an additional group (different universities—DU) was created, which comprised 6.0% of the total sample.

Polish universities vary with respect to their size and educational profile. There are relatively few large universities with over 20 thousand people (roughly 20 out of 400). Most universities are of medium or small size. The largest university in Poland is the University of Warsaw. It also has one of the broadest selections of programs. A characteristic aspect of Polish universities is that they offer specific educational profiles, for example, universities focusing on medical education. Another example is the Pomeranian University, which specializes in teaching education. Table 2 shows basic characteristics of universities which were widely represented in the research sample [45–50].

**Table 2:** Characteristics of the universities most widely represented in the current sample.

		UG	UAM	UW	UJK	AP
**Number (in thousands)**	Students	24	37	43	10	3
	Teachers	1.8	2.8	3.8	0.9	0.37
	Study programs	89	80	100	50	27
**Home city of the university (in thousands)**		470	534	1 790	194	90

In the current study, women represented 89.6% of the sample. Participants between 21 and 24 years of age and those living in large cities represented the largest group (59.5% and 36.7%, respectively) Over 80% of the participants were full-time students, which reflected the general population distribution of students in Poland [51]. Undergraduate students also represented a larger group.

### Measures

The multidimensional COPE inventory by Carver et al [52, 53] is one of the most popular measures of strategies of coping with stress. It can be used to measure dispositional (typical) and situational coping. The Mini-COPE inventory in a Polish adaptation by Juczyński and Ogińska-Bulik [54] was used in the current study. The internal consistency of the Polish version of the Mini-COPE was estimated based on a sample of 200 people aged between 25 and 60. The split-half reliability was 0.86 (Guttman’s coefficient = 0.87). The repeatability was satisfactory for the majority of the scales. The Polish version of the Mini-COPE comprises 28 items, which form 14 coping strategies. It is used to measure typical reactions in situations of intense stress. The main question is: What do you usually do when you are stressed by a problem? The coping strategies are described in statements such as “I work or do other things in order not to think about the problem.” Each statement is graded on a four-point Likert scale: 1 = very seldom, 2 = fairly seldom, 3 = fairly often, 4 = very often. Each of the 14 coping strategies is measured by two items.

**Fig 1:**
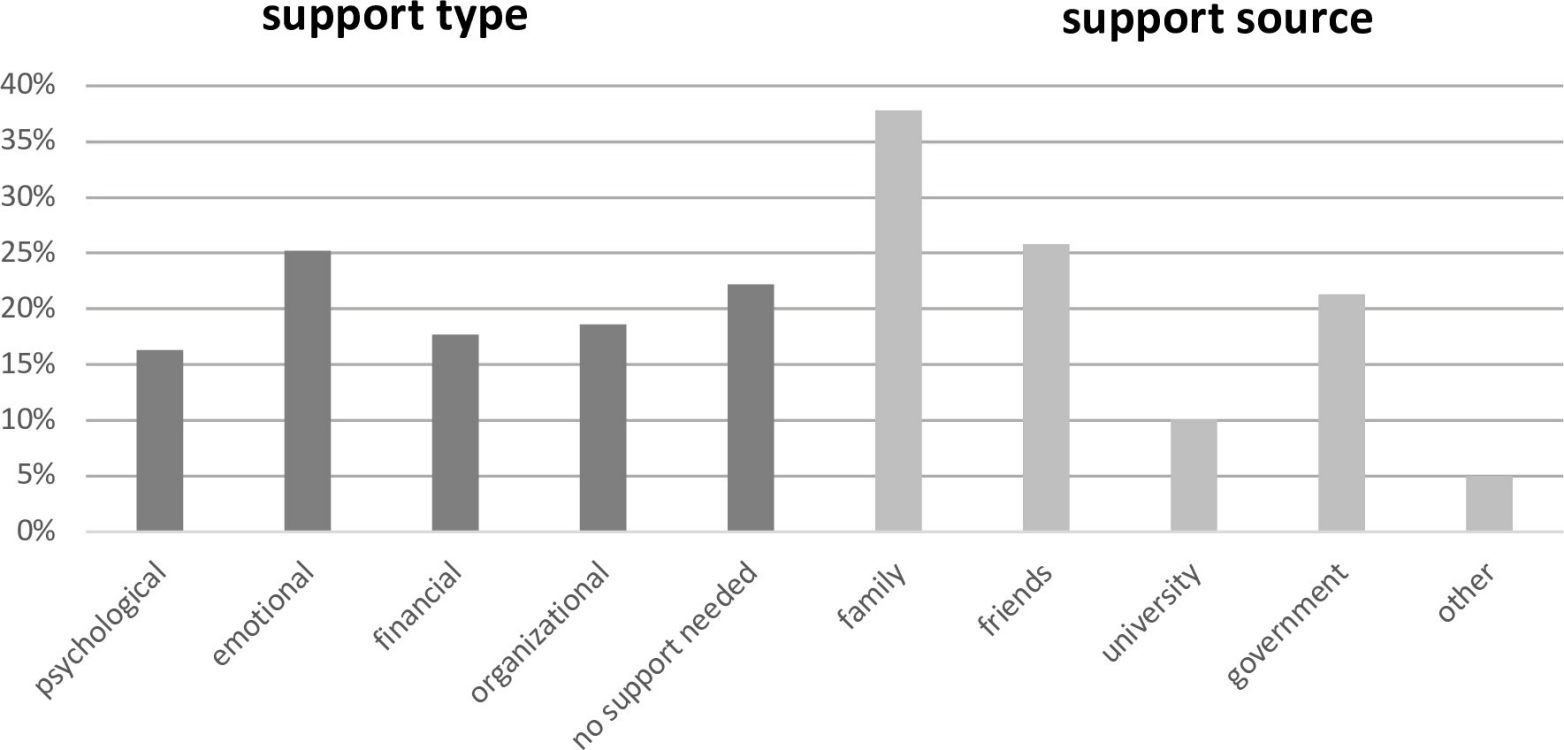
Support expected during the pandemic: Type and source.

The Mini-COPE inventory was supplemented with two other semi-open questions. The first concerned the type of support the students expected during the pandemic. The available answers were: psychological, emotional, financial, organizational support, no support needed, and other (to be filled out by the students if necessary). The second supplemental question concerned the sources from which the students expected support during the pandemic. In this case, the students could select the closest appropriate answer from among: family, friends, the university, the government, and other (to be filled out by the students if necessary). Fig 1 shows the distribution of the students’ answers to the supplementary questions about support.

Regarding the source of support, 5.0% of the students chose “other.” This category comprised the following answers: significant other (0.9%), nobody (1.2%), psychologist/therapist (0.5%), myself (1.4%), all of the above (0.3%), and other combinations indicating two sources, for example, family and the government (0.7%).

### Analyses

Statistical analyses were carried out using the IBM SPSS Statistics 25.0 software. The program was used to calculate basic descriptive statistics together with the Kolmogorov-Smirnov test of normality. Additionally, the Cronbach’s α coefficient was used to calculate the reliability of the Mini-COPE scales. To compare coping strategies between two groups, Mann-Whitney’s *U* test was used. To compare a higher number of groups, a one-way analysis of variance (ANOVA) was used, and if variance was not equal between the groups, Welch’s correction was additionally applied. Tukey’s HSD test (if variance was homogenous) or Dunnett’s T3 test (if variance was heterogenous) was used for post hoc analyses. To estimate intergroup differences in coping strategies, Pearson’s *r* correlation analysis was carried out. The significance level was set at α = 0.05. In order to distinguish the groups of participants in terms of coping strategies, a two-step cluster analysis was carried out.

## Results

### Students’ dominant strategies of coping with stress during the pandemic

Based on the descriptive statistics and the results of the Kolmogorov-Smirnov test of normality, it was concluded that neither of the analyzed variables assumed a distribution close to the Gaussian curve. Skewness values were within the <-2;2> range, which means that it was not significant [55]. Additionally, the Cronbach’s α coefficient was used to calculate the reliability of the Mini-COPE scales. The analysis showed satisfactory reliability for most of the scales. Relatively low reliability was obtained for the scales of acceptance, humor, self-distraction, and venting of emotions. Detailed results are shown in Table 3.

**Table 3:** Basic descriptive statistics with the Kolmogorov-Smirnov test of normality and reliability (Cronbach’s α).

	*M*	*Me*	*SD*	*Sk*.	*Kurt*.	*Min*.	*Max*.	*D*	*p*	*α*
Active coping	1.79	2.00	0.68	-0.32	-0.10	0.00	3.00	0.22	<0.001	0.73
Planning	1.90	2.00	0.65	-0.36	0.01	0.00	3.00	0.22	<0.001	0.64
Positive reframing	1.73	2.00	0.71	-0.47	-0.08	0.00	3.00	0.25	<0.001	0.78
Acceptance	2.05	2.00	0.58	-0.39	0.44	0.00	3.00	0.22	<0.001	0.59
Humor	1.08	1.00	0.65	0.25	-0.27	0.00	3.00	0.16	<0.001	0.50
Religious coping	0.89	0.50	0.97	0.76	-0.63	0.00	3.00	0.24	<0.001	0.90
Seeking emotional support	1.85	2.00	0.80	-0.45	-0.32	0.00	3.00	0.20	<0.001	0.83
Seeking instrumental support	1.69	2.00	0.80	-0.35	-0.47	0.00	3.00	0.19	<0.001	0.75
Self-distraction	1.75	2.00	0.67	-0.37	-0.04	0.00	3.00	0.18	<0.001	0.42
Denial	0.73	0.50	0.68	0.83	0.21	0.00	3.00	0.19	<0.001	0.61
Venting emotions	1.57	1.50	0.65	-0.20	-0.16	0.00	3.00	0.16	<0.001	0.24
Substance use	0.50	0.00	0.71	1.40	1.27	0.00	3.00	0.35	<0.001	0.93
Behavioral disengagement	0.87	1.00	0.64	0.53	-0.14	0.00	3.00	0.20	<0.001	0.70
Self-blame	1.24	1.00	0.84	0.35	-0.65	0.00	3.00	0.17	<0.001	0.67

The dominant coping strategies among Polish students were: acceptance, planning, and seeking emotional support. The least frequent strategies were: substance use, denial, behavioral disengagement, and religious coping.

Pearson’s correlation analysis was used to examine the relationships between individual coping strategies in the current sample (Table 4). Active coping was positively correlated with the following coping strategies: planning (strong correlation), positive reframing, religious coping, emotional support seeking, instrumental support seeking, self-distraction, venting of emotions, and self-blame (weak correlations). The higher the frequency of active coping, the higher the frequencies of the above strategies as well. Active coping was moderately and negatively correlated with behavioral disengagement, which means that the higher the frequency of active coping, the lower the frequency of behavioral disengagement.

**Table 4:** Pearson’s correlation coefficients between the strategies of coping with stress.

	1	2	3	4	5	6	7	8	9	10	11	12	13	14
1. Active coping	-													
2. Planning	0.60**	-												
3. Positive reframing	0.22**	0.31**	-											
4. Acceptance	0.06	0.24**	0.32**	-										
5. Humor	-0.01	0.05	0.28**	0.23**	-									
6. Religious coping	0.11**	0.14**	0.21**	-0.01	-0.01	-								
7. Seeking emotional support	0.21**	0.21**	0.24**	0.07	0.11*	0.21**	-							
8. Seeking instrumental support	0.26**	0.30**	0.17**	0.01	0.08	0.22**	0.75**	-						
9. Self-distraction	0.11**	0.14**	0.10*	0.02	0.11**	0.10*	0.11*	0.17**	-					
10. Denial	-0.05	-0.08*	-0.01	-0.22**	0.07	0.06	0.04	0.06	0.25**	-				
11. Venting of emotions	0.10*	0.16**	-0.01	-0.02	0.05	0.11*	0.28**	0.38**	0.33**	0.25**	-			
12. Substance use	-0.07	-0.07	<-0.01	-0.03	0.17**	-0.13**	-0.01	0.02	0.13**	0.17**	0.14**	-		
13. Behavioral disengagement	-0.34**	-0.32**	-0.27**	-0.20**	0.03	0.06	-0.13**	-0.02	0.12**	0.23**	0.18**	0.18**	-	
14. Self-blame	0.15**	0.07	-0.17**	-0.23**	-0.06	0.02	<0.01	0.13**	0.19**	0.15**	0.27**	0.14**	0.37**	-

*p < 0.05;

**p < 0.01.

Another coping strategy—planning—was positively and weakly-to-moderately correlated with positive reframing, acceptance, religious coping, emotional support seeking, instrumental support seeking, self-distraction, and self-blame. A weak, negative correlation occurred between planning and denial, and a moderate one between behavioral activation—the higher the frequency of planning, the lower the frequency of denial and behavioral disengagement.

Positive reframing was positively and weakly-to-moderately correlated with acceptance, humor, religious coping, emotional support seeking, instrumental support seeking, and self-distraction. This coping strategy was also weakly and negatively correlated with behavioral disengagement and self-blame.

Acceptance was weakly and positively correlated with humor and negatively with denial, behavioral disengagement, and self-blame. In turn, humor was weakly and positively correlated with emotional support seeking, self-distraction, and substance use. A weak and positive correlation also occurred between religious coping and emotional support seeking, instrumental support seeking, and venting of emotions. Religious coping was also weakly and negatively correlated with substance use.

Another strategy—emotional support seeking—was strongly and positively correlated with instrumental support seeking. A weak and positive correlation occurred between the following coping strategies: self-distraction and venting of emotions. Emotional support seeking was weakly and negatively correlated with behavioral disengagement.

On the other hand, seeking instrumental support was weakly-to-moderately and positively correlated with self-distraction, venting of emotions, and self-blame.

Self-distraction was weakly-to-moderately and positively correlated with denial, venting of emotions, substance use, behavioral disengagement, and self-blame.

Denial, venting of emotions, substance use, behavioral disengagement, and self-blame were positively correlated with each other on a weak-to-moderate level (the relationship between behavioral disengagement and self-blame).

The remaining correlations between the coping strategies were not statistically significant.

### Students’ strategies of coping with stress–cluster analysis

**Fig 2:**
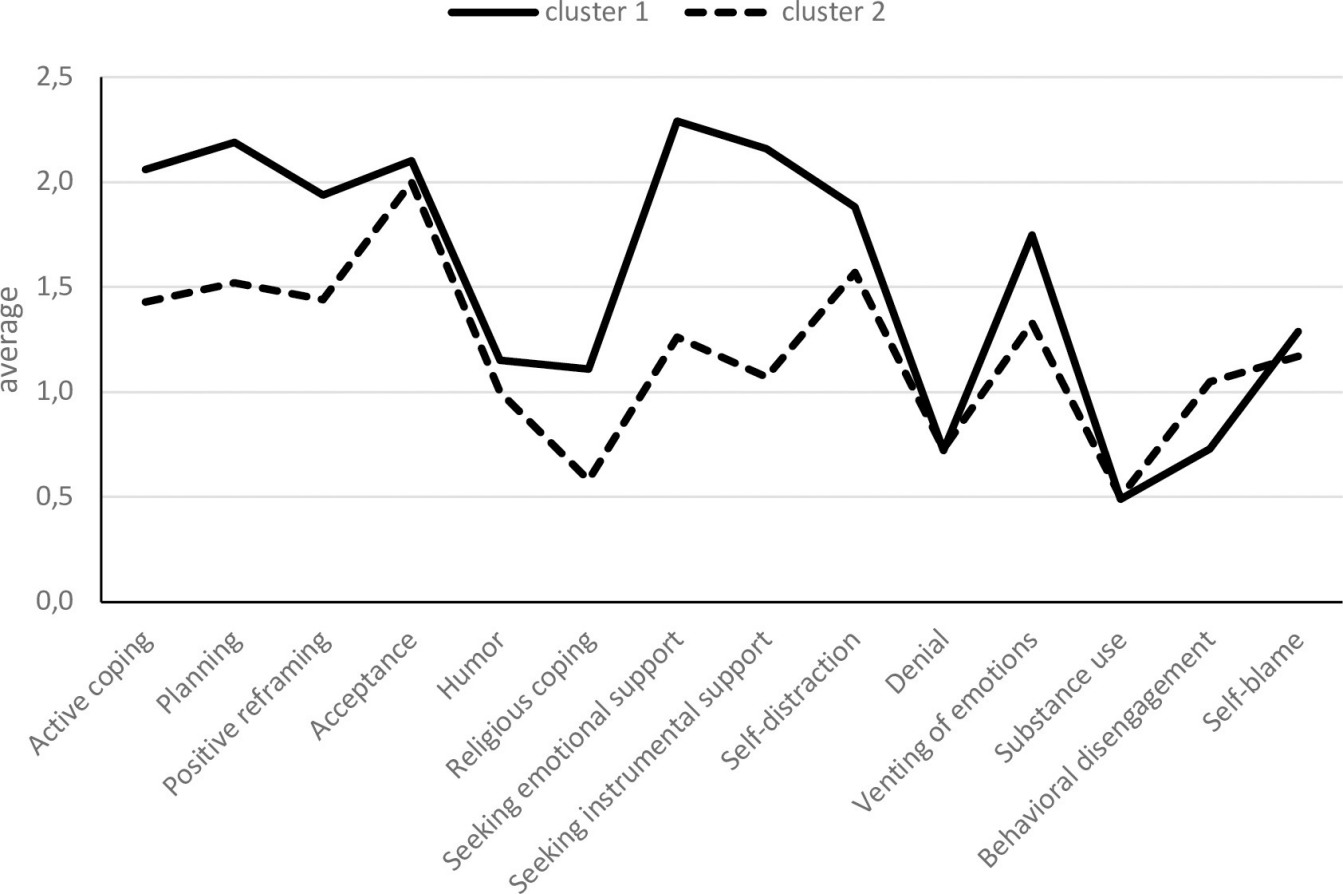
Cluster analysis for strategies of coping with stress.

To distinguish groups of participants based on their coping strategies, a two-step cluster analysis was carried out. It allowed for distinguishing two clusters (Fig 2) for which the silhouette value was 0.2, indicating a satisfactory quality of clustering. From among the coping strategies included in the model, the most important ones were: seeking instrumental support, seeking emotional support, and planning. These strategies differentiated the two clusters to the highest degree. The least important strategies were substance use, denial, and self-blame.

Table 5 shows the comparison of the clusters with regard to the analyzed strategies. The analysis showed no statistically significant differences for denial, substance use, and self-blame. Differences for other coping strategies were statistically significant, with Cluster 1 participants scoring higher on active coping, planning, positive reframing, humor, religious coping, seeking emotional and instrumental support, self-distraction, and venting of emotions, and lower on behavioral disengagement compared to Cluster 2 participants.

**Table 5:** Comparisons of coping strategies between the clusters.

Strategies	1 (*n* = 331)		2 (*n* = 246)				*95% CI*		
	*M*	*SD*	*M*	*SD*	*t*	*p*	*LL*	*UL*	*Cohen’s d*
Active coping	2.06	0.54	1.43	0.67	12.23	<0.001	0.54	0.74	1.06
Planning	2.19	0.48	1.52	0.65	13.40	<0.001	0.57	0.76	1.18
Positive reframing	1.94	0.58	1.44	0.77	8.39	<0.001	0.38	0.61	0.74
Acceptance	2.10	0.54	2.00	0.63	2.04	0.042	0.00	0.19	0.17
Humor	1.15	0.59	1.00	0.72	2.54	0.012	0.03	0.25	0.22
Religious coping	1.11	1.02	0.58	0.80	7.01	<0.001	0.38	0.68	0.57
Seeking emotional support	2.29	0.53	1.26	0.71	19.15	<0.001	0.92	1.13	1.68
Seeking instrumental support	2.16	0.52	1.07	0.67	21.33	<0.001	0.99	1.19	1.86
Self-distraction	1.88	0.61	1.57	0.71	5.55	<0.001	0.20	0.42	0.48
Denial	0.72	0.67	0.73	0.69	-0.11	0.915	-0.12	0.11	0.01
Venting of emotions	1.75	0.56	1.33	0.69	7.84	<0.001	0.32	0.53	0.68
Substance use	0.49	0.68	0.50	0.75	-0.07	0.947	-0.12	0.11	0.01
Behavioral disengagement	0.73	0.53	1.05	0.73	-5.84	<0.001	-0.43	-0.21	0.51
Self-blame	1.29	0.80	1.17	0.89	1.71	0.088	-0.02	0.26	0.14

### Sociodemographic factors and strategies of coping with stress

To estimate the gender differences in coping strategies, Mann-Whitney’s *U* test was used. The analysis showed statistically significant gender differences for humor, emotional support seeking, instrumental support seeking, self-distraction, denial, and venting of emotions. Men in the current sample reported using humor significantly more often than women, but they reported using religious coping, emotional support seeking, instrumental support seeking, self-distraction, and denial less frequently. The results of the analysis are shown in Table 6.

**Table 6:** Gender differences in strategies of coping with stress.

	Women (*n* = 517)			Men (*n* = 60)					
Strategies	Mean range	*Me*	*IQR*	Mean range	*Me*	*IQR*	*Z*	*p*	*R*
Active coping	287.42	2.00	0.50	302.63	2.00	1.00	-0.69	0.489	0.03
Planning	286.31	2.00	1.00	312.16	2.00	1.00	-1.18	0.237	0.05
Positive reframing	291.95	2.00	1.00	263.55	2.00	1.00	-1.30	0.192	0.05
Acceptance	288.20	2.00	0.50	295.88	2.00	1.00	-0.35	0.723	0.01
Humor	281.60	1.00	1.00	352.73	1.50	1.00	-3.21	0.001	0.13
Religious coping	293.75	0.50	1.50	248.07	0.00	1.00	-2.10	0.036	0.09
Seeking emotional support	299.14	2.00	1.00	201.66	1.50	1.00	-4.40	<0.001	0.18
Seeking instrumental support	298.19	2.00	1.00	209.79	1.50	1.50	-3.98	<0.001	0.17
Self-distraction	294.70	2.00	0.50	239.89	1.50	1.00	-2.48	0.013	0.10
Denial	294.12	0.50	1.00	244.91	0.50	1.00	-2.23	0.025	0.09
Venting of emotions	293.67	1.50	1.00	248.80	1.50	1.00	-2.03	0.042	0.08
Substance use	286.80	0.00	1.00	307.94	0.00	1.00	-1.05	0.293	0.04
Behavioral disengagement	288.31	1.00	0.50	294.95	1.00	1.38	-0.30	0.763	0.01
Self-blame	286.49	1.00	1.50	310.60	1.50	1.50	-1.08	0.281	0.04

Gender differences could result from differences in gender role socialization [56, 57]. Women are socialized to be more emotional and seek support in interpersonal relationships. On the other hand, men are socialized to cope with their problems on their own or use humor.

Using a one-way analysis of variance (ANOVA), coping strategy use was compared between age groups (Table 7). The analysis showed statistically significant differences for six strategies: active coping, planning, positive reframing, venting of emotions, behavioral disengagement, and self-blame. To estimate the character of the intergroup differences, an additional post hoc analysis using Tukey’s HSD test was carried out when the variance was equal between the groups, and Dunnett’s T3 test, when the variance was unequal. This type of post hoc analysis was used due to the disproportions in the size of the compared groups. In the case of unequal variances, the Welch correction was also applied.

**Table 7:** One-way analysis of variance of differences in strategies of coping with stress between age groups.

	18–20 (*n =* 143)		21–24 (*n =* 343)		25–30 (*n =* 56)		31 and over (*n =* 35)				
	*M*	*SD*	*M*	*SD*	*M*	*SD*	*M*	*SD*	*F*	*p*	η^2^
Active coping	1.71	0.73	1.77	0.65	2.00	0.69	2.06	0.64	4.47	0.004	0.02
Planning	1.86	0.70	1.86	0.65	2.11	0.52	2.17	0.58	5.78^a^	0.001	0.02
Positive reframing	1.73	0.74	1.67	0.72	1.80	0.62	2.16	0.45	11.02^a^	<0.001	0.03
Acceptance	2.06	0.58	2.04	0.60	2.04	0.45	2.16	0.63	0.42	0.737	0.00
Humor	1.07	0.67	1.11	0.67	1.03	0.56	0.96	0.59	0.79	0.502	0.00
Religious coping	0.93	1.01	0.84	0.94	0.98	1.03	1.04	0.96	0.88	0.450	0.00
Seeking emotional support	1.91	0.82	1.86	0.78	1.80	0.74	1.57	0.90	1.80	0.145	0.01
Seeking instrumental support	1.77	0.80	1.69	0.80	1.68	0.68	1.40	0.86	2.07	0.103	0.01
Self-distraction	1.81	0.69	1.75	0.66	1.71	0.67	1.47	0.66	2.43	0.064	0.01
Denial	0.80	0.70	0.73	0.69	0.64	0.62	0.54	0.55	1.70	0.164	0.01
Venting of emotions	1.59	0.62	1.61	0.68	1.54	0.55	1.20	0.55	4.38	0.004	0.02
Substance use	0.50	0.73	0.50	0.72	0.54	0.73	0.34	0.53	0.60	0.612	0.00
Behavioral disengagement	0.96	0.68	0.87	0.61	0.75	0.65	0.60	0.62	3.76	0.010	0.02
Self-blame	1.28	0.84	1.24	0.84	1.38	0.84	0.86	0.80	3.12	0.025	0.02

*—Welch’s correction was applied.

18-20-year-olds reported statistically significantly less frequent active coping than did 25-30-year-olds (*p* = 0.032) and those 31 and over (*p =* 0.032). The youngest participants also reported less frequent planning than did 25-30-year-olds (*p =* 0.039) and those 31 and over (*p =* 0.045), while 21-24-year-olds reported significantly less frequent planning than 25-30-year-olds (*p =* 0.015) and those 31 and over (*p =* 0.030). The coping strategy of positive reframing was more frequent in the oldest group compared to the younger groups (p ≤ 0.013). Those 31 and over also reported significantly less frequent venting of emotions compared to 18-20-year-olds (*p =* 0.007) and 21-24-year-olds (*p =* 0.002). Behavioral disengagement differed significantly between the youngest and the oldest group (*p =* 0.014), with the higher frequency of this strategy being reported in the 18-20-year-olds group. Those 31 and over reported less frequent self-blame than did 18-20-year-olds (*p =* 0.036) and 25-30-year-olds (*p =* 0.019).

The current data show that the oldest students used active coping strategies more often during the pandemic than did the younger students. The aim of these strategies is to solve the problem causing difficult internal tension rather than to avoid the situation altogether. This effect may be related to the older students having greater life experience, including academic experience, at 31 years of age.

In the next step, differences in coping strategy use depending on the place of residence were examined (Table 8). To this end, a one-factor ANOVA was carried out. It showed significant intergroup differences for the following coping strategies: active coping, planning, humor, religious coping, denial, and substance use. Participants living in cities with over 100 thousand residents reported using planning significantly more often than those living in villages (*p <* 0.001) or towns up to 20 thousand residents (*p =* 0.006).

**Table 8:** One-factor analysis of variance of differences in strategies of coping with stress depending on place of residence.

	Village (*n* = 202)		Town up to 20 thousand residents (*n =* 50)		Town with between 20 and 100 thousand residents residents (*n =* 113)		City with over 100 thousand residents (*n =* 212)				
	*M*	*SD*	*M*	*SD*	*M*	*SD*	*M*	*SD*	*F*	*p*	η^2^
Active coping	1.75	0.67	1.67	0.65	1.73	0.71	1.90	0.67	2.80	0.039	0.01
Planning	1.80	0.66	1.72	0.63	1.90	0.61	2.05	0.65	6.92	<0.001	0.03
Positive reframing	1.71	0.68	1.69	0.87	1.69	0.73	1.77	0.70	0.45	0.714	0.00
Acceptance	2.02	0.59	2.01	0.60	2.04	0.56	2.09	0.58	0.62	0.604	0.00
Humor	1.09	0.67	0.88	0.57	1.00	0.61	1.17	0.67	3.72	0.011	0.02
Religious coping	1.05	1.00	1.09	0.98	0.82	0.97	0.72	0.91	4.83	0.002	0.02
Seeking emotional support	1.75	0.83	2.02	0.78	1.83	0.82	1.91	0.75	2.15	0.092	0.01
Seeking instrumental support	1.60	0.79	1.67	0.80	1.69	0.85	1.79	0.77	2.11	0.097	0.01
Self-distraction	1.72	0.70	1.61	0.66	1.81	0.63	1.77	0.66	1.24	0.293	0.01
Denial	0.83	0.68	0.74	0.71	0.80	0.71	0.59	0.64	4.98	0.002	0.03
Venting of emotions	1.50	0.64	1.67	0.70	1.57	0.69	1.63	0.63	1.61	0.185	0.01
Substance use	0.37	0.59	0.37	0.61	0.57	0.78	0.61	0.78	5.32^a^	0.002	0.03
Behavioral disengagement	0.86	0.59	0.92	0.63	0.93	0.65	0.82	0.68	0.84	0.471	0.00
Self-blame	1.17	0.83	1.12	0.86	1.33	0.86	1.29	0.84	1.55	0.201	0.01

*—Welch’s correction was applied.

Participants living in towns up to 20 thousand residents reported using humor significantly less frequently than those living in cities with over 100 thousand residents (*p =* 0.021). Participants living in villages reported using religious coping significantly more often than those living in cities with over 100 thousand residents (*p =* 0.004). This is related to a more traditional upbringing and culture in Polish rural regions, where religious rituals play a significant role. Participants living in cities with over 100 thousand residents reported using denial significantly less frequently than those living in villages (*p =* 0.002) and in towns with between 20 and 100 thousand residents (*p =* 0.034). Substance use was reported more frequently among participants living in biggest cities compared to participants living in villages (*p =* 0.002). This is because various psychoactive substances are more easily available in large cities.

After the correction for multiple comparisons was applied, a post hoc analysis using Tukey’s HSD test did not reveal statistically significant intergroup differences in active coping.

To compare full-time and extramural students’ use of coping strategies, Mann-Whitney’s *U* test was used. It showed that extramural students reported using active coping and positive reframing more frequently, and humor, instrumental support seeking, self-distraction, venting of emotions, substance use, and self-blame less frequently compared to full-time students. The results of the analysis are presented in Table 9.

**Table 9:** Comparison of coping strategy use between full-time and extramural students.

	Full-time students (*n* = 450)			Extramural students (*n* = 127)					
	Mean range	*Me*	*IQR*	Mean range	*Me*	*IQR*	*Z*	*p*	*R*
Active coping	281.20	2.00	0.50	316.65	2.00	1.00	-2.19	0.028	0.09
Planning	284.05	2.00	1.00	306.55	2.00	1.00	-1.40	0.162	0.06
Positive reframing	281.72	2.00	1.00	314.78	2.00	0.50	-2.06	0.039	0.09
Acceptance	286.56	2.00	1.00	297.64	2.00	0.50	-0.69	0.488	0.03
Humor	296.47	1.00	1.00	262.52	1.00	1.00	-2.08	0.037	0.09
Religious coping	286.43	0.50	1.50	298.12	1.00	2.00	-0.73	0.466	0.03
Seeking emotional support	294.50	2.00	1.00	269.51	2.00	1.00	-1.53	0.126	0.06
Seeking instrumental support	296.36	2.00	1.00	262.93	1.50	1.00	-2.04	0.041	0.08
Self-distraction	298.44	2.00	0.50	255.54	1.50	1.00	-2.64	0.008	0.11
Denial	291.85	0.50	1.00	278.89	0.50	1.00	-0.80	0.424	0.03
Venting of emotions	299.50	1.50	1.00	251.81	1.50	1.00	-2.93	0.003	0.12
Substance use	295.81	0.00	1.00	264.87	0.00	1.00	-2.09	0.037	0.09
Behavioral disengagement	294.64	1.00	0.50	269.02	1.00	0.50	-1.58	0.114	0.07
Self-blame	297.86	1.00	1.50	257.60	1.00	1.00	-2.44	0.015	0.10

**Fig 3:**
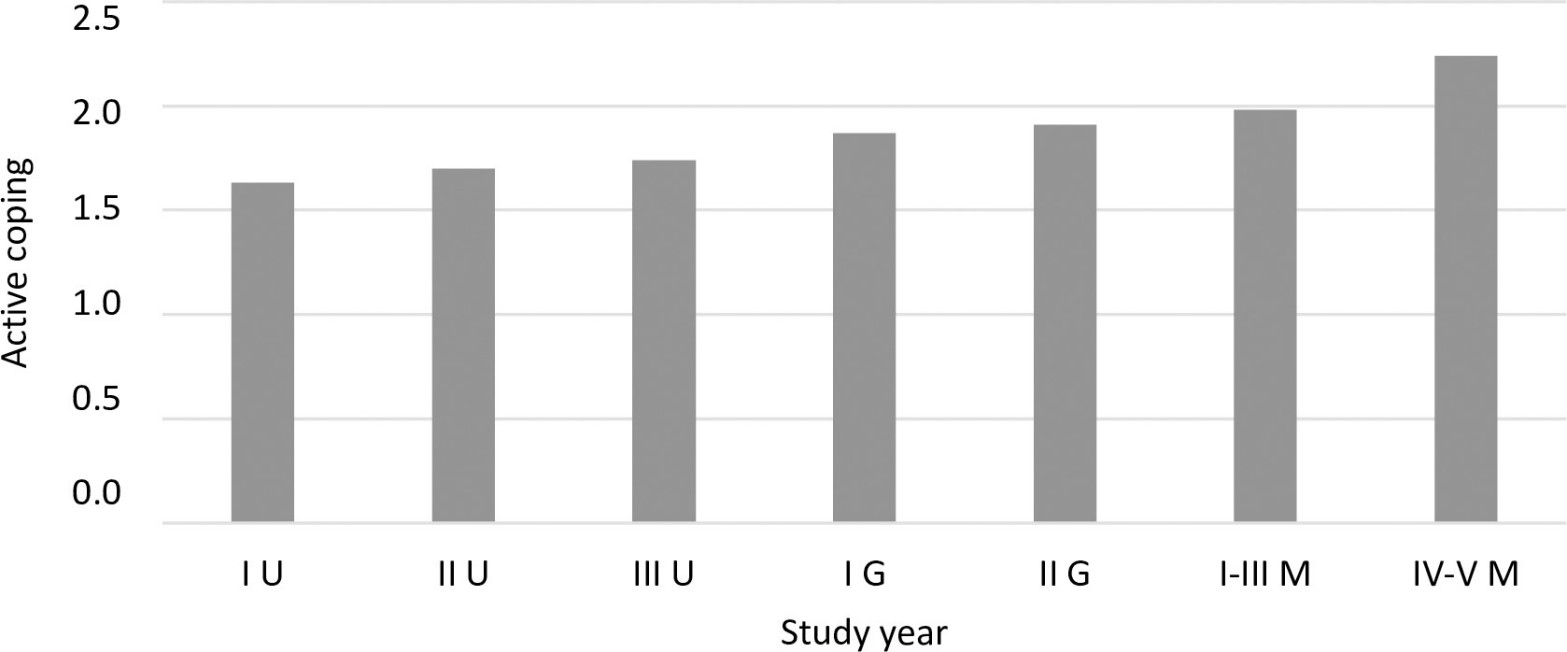
The coping strategy of active coping (mean) among students in individual program years (F = 5.72, p<0.001, eta = 0.06). I U–I Undergraduate (n = 142), II U–II Undergraduate (n = 100), III U–III Undergraduate (n = 104), I G–I Graduate (n = 66), II G–II Graduate (n = 70), I-III M–I-III uniform Master’s studies (n = 64), IV-V M–IV-V uniform Master’s studies (n = 31).

**Fig 4:**
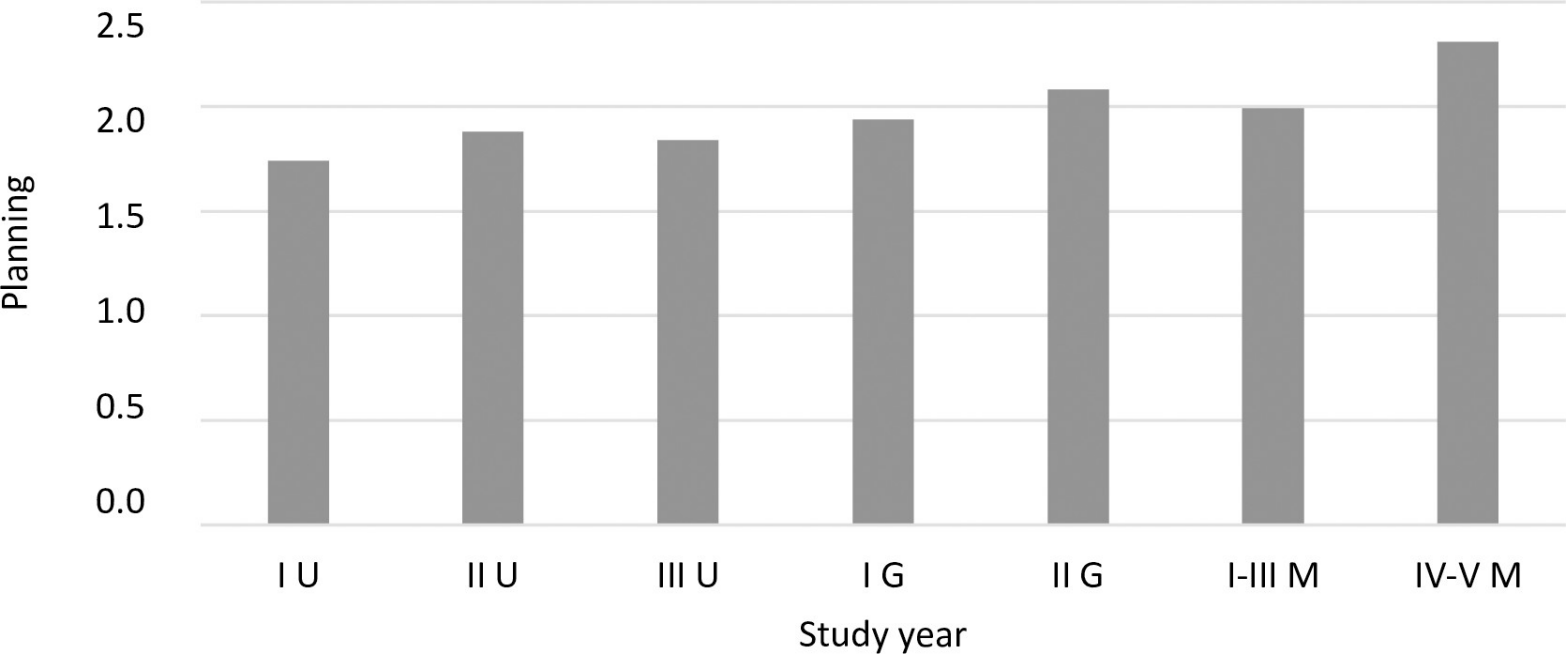
The coping strategy of planning (mean) among students in individual program years (F = 4.87, p<0.001, eta = 0.05).

**Fig 5:**
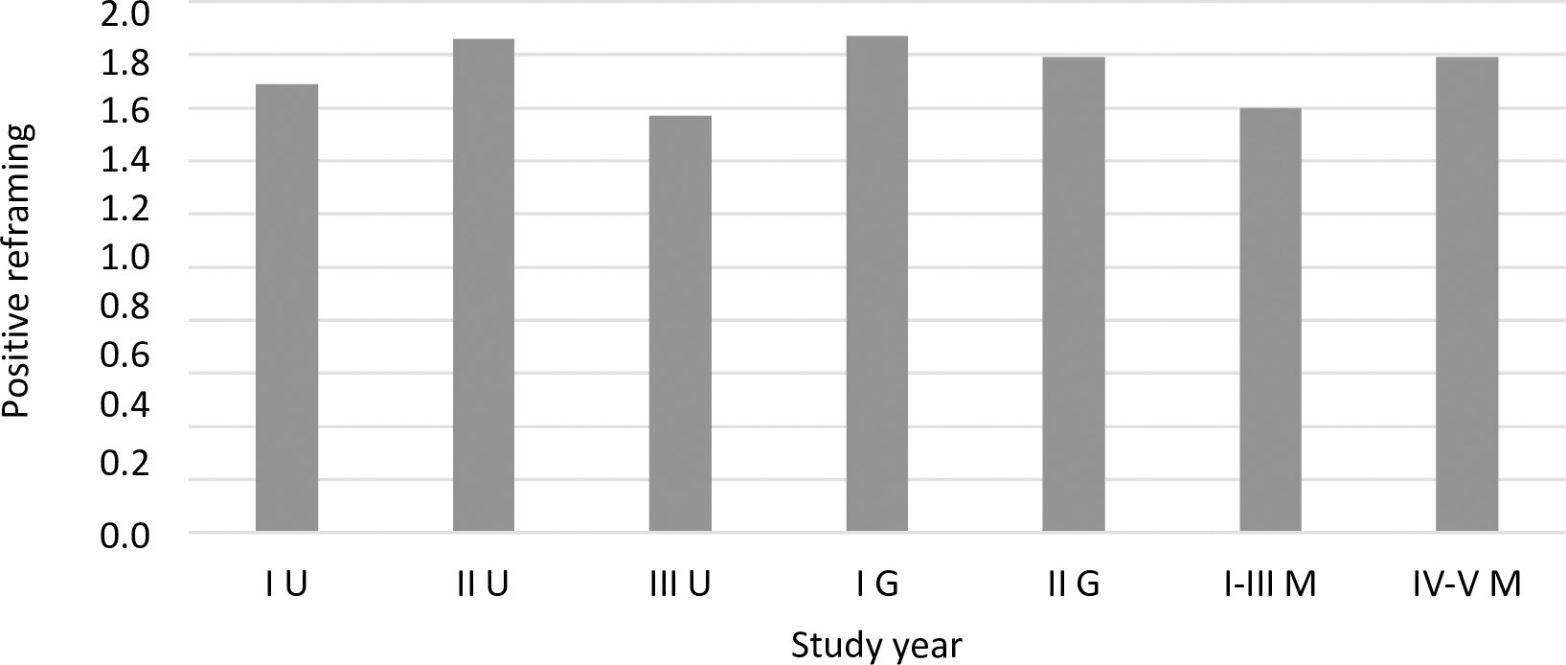
The coping strategy of positive reframing (mean) among students in individual program years (F = 2.51^a^, p = 0.023, eta = 0.01). * Welch’s correction was applied.

**Fig 6:**
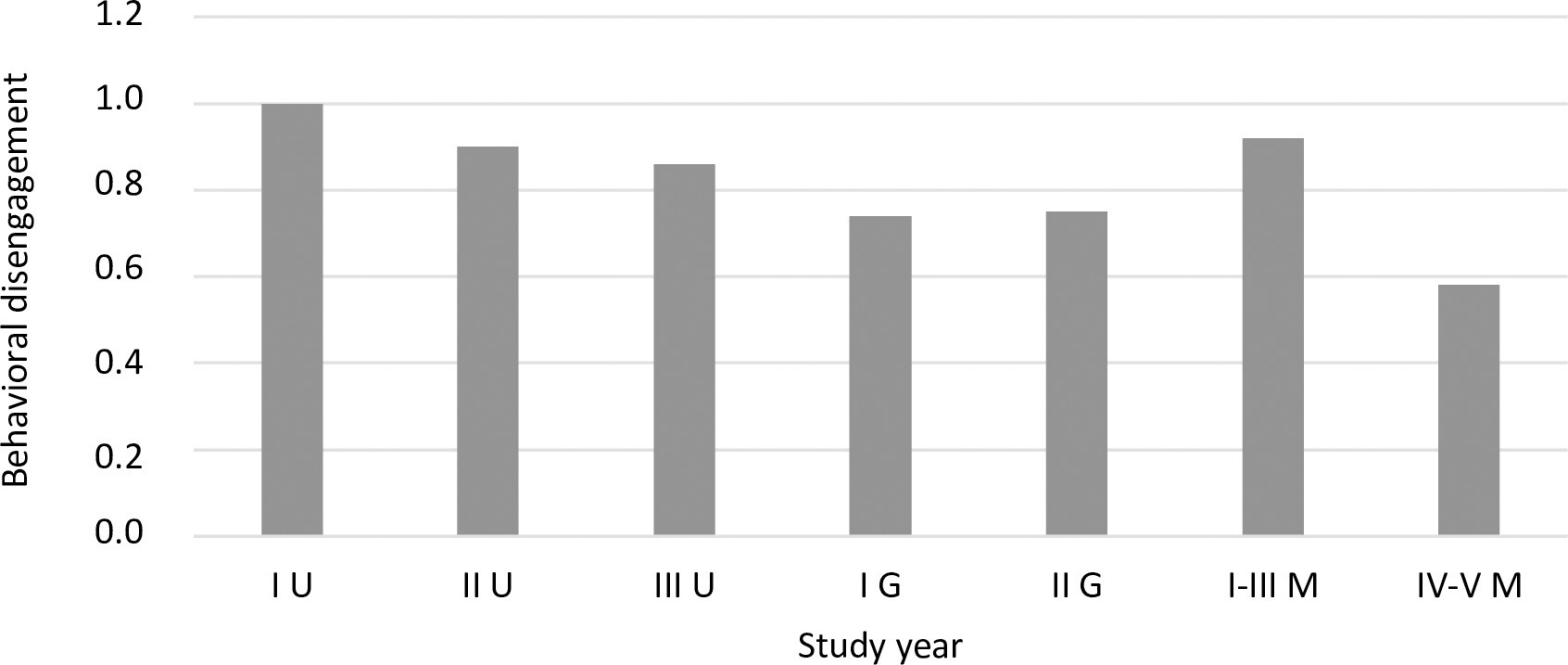
The coping strategy of behavioral disengagement (mean) among students in individual program years (F = 3.06, p = 0.006, eta = 0.03).

Using a one-factor ANOVA, coping strategies were compared between students in different program years. The analysis showed significant differences for four strategies: active coping (Fig 3), planning (Fig 4), positive reframing (Fig 5), and behavioral disengagement (Fig 6).

A detailed post hoc analysis showed that first year undergraduate students reported using active coping less frequently compared to second year graduate students (*p =* 0.048), first-third year uniform Master’s students (*p =* 0.009), and fourth-fifth year uniform Master’s students (*p <* 0.001). Second year undergraduate students also reported using active coping less frequently than did fourth-fifth year uniform Master’s students (*p =* 0.001), similar to third year undergraduate students (*p =* 0.004). First year undergraduate students reported using planning less frequently compared to second year graduate students (*p =* 0.006) and fourth-fifth year uniform Master’s students (*p <* 0.001). Fourth-fifth year uniform Master’s students reported using planning more frequently than second year (*p =* 0.018) and third year (*p =* 0.007) undergraduate students. A significant difference in the frequency of using behavioral disengagement occurred between first year undergraduate students and fourth-fifth year uniform Master’s students (*p =* 0.015). First year undergraduate students reported using behavioral disengagement more frequently than did fourth-fifth year uniform Master’s students. For positive reframing, after applying the correction for multiple comparisons, Dunnett’s T3 test did not show statistically significant intergroup differences. A statistical trend (*p =* 0.053) was observed between third year undergraduate students and first year graduate students—first year graduate students reported a slightly higher frequency of using positive reframing than did third year undergraduate students.

### Students’ strategies of coping with stress and the type and source of needed social support

A one-way ANOVA was used to estimate the differences in coping strategy use depending on the need for a given type of social support (Table 10). The post hoc analysis showed that participants who indicated a need for psychological support reported using the coping strategy of positive reframing less frequently than those who did not indicate any need for support (*p =* 0.027). Also, those who indicated a need for financial support reported using positive reframing less frequently than those who did not indicate any need for support (*p =* 0.034). Those who did not indicate any need for support used the coping strategy of acceptance more frequently than those who indicated a need for psychological (*p =* 0.013) and emotional (*p =* 0.002) support. This is due to the fact that these individuals cope with the pandemic-related difficulties on their own. Those who indicated a need for financial support also used religious coping less frequently than those who indicated a need for emotional support (*p =* 0.007). Participants who indicated a need for emotional support reported using the coping strategy of emotional support seeking more frequently than those who indicated a need for financial support (*p =* 0.004) and those who did not indicate any need for support (*p =* 0.034). Analogous differences were observed for instrumental support seeking. Participants who indicated a need for emotional support reported using this coping strategy more often than did those who indicated a need for financial support (*p <* 0.001) and those who did not indicate any need for support (*p =* 0.001). Those who did not indicate any need for support reported using self-distraction less frequently than those who indicated needing psychological (*p =* 0.014) and emotional (*p =* 0.003) support. Also, participants who did not indicate any need for support reported using the coping strategy of denial less frequently than did those who indicated a need for psychological (*p =* 0.002) or emotional (*p =* 0.002) support. Participants who did not indicate any need for support reported using venting of emotions less frequently than did those who indicated a need for psychological (*p <* 0.001) and emotional (*p <* 0.001) support, whereas participants who indicated a need for financial support reported using venting of emotions less frequently than did those who indicated a need for emotional support (*p =* 0.003). Participants who indicated a need for psychological support reported more frequent substance use than did those who did not indicate any need for support (*p =* 0.002). Participants also used behavioral disengagement more often than did those who indicated a need for financial (*p =* 0.001) and organizational support (*p =* 0.001), or did not indicate any need for support (*p <* 0.001). In turn, those participants who indicated a need for emotional support reported using behavioral disengagement more frequently than those who did not indicate any need for support at all (*p =* 0.006).

**Table 10:** One-factor analysis of variance of differences in coping strategy use depending on the type of support needed.

Coping strategy	Type of support	*N*	*M*	*SD*	*F*	*p*	η^2^
Active coping	Psychological	94	1.76	0.64	1.24	0.293	0.01
	Emotional	145	1.80	0.67			
	Financial	102	1.70	0.72			
	Organizational	107	1.80	0.66			
	No support needed	128	1.89	0.69			
Planning	Psychological	94	1.88	0.60	1.16^a^	0.328	0.01
	Emotional	145	1.96	0.60			
	Financial	102	1.78	0.77			
	Organizational	107	1.90	0.60			
	No support needed	128	1.96	0.68			
Positive reframing	Psychological	94	1.58	0.72	3.66	0.006	0.03
	Emotional	145	1.72	0.72			
	Financial	102	1.59	0.70			
	Organizational	107	1.83	0.69			
	No support needed	128	1.86	0.71			
Acceptance	Psychological	94	1.95	0.57	4.94	0.001	0.03
	Emotional	145	1.94	0.57			
	Financial	102	2.15	0.60			
	Organizational	107	2.05	0.56			
	No support needed	128	2.20	0.57			
Humor	Psychological	94	0.99	0.70	0.82	0.513	0.01
	Emotional	145	1.07	0.64			
	Financial	102	1.14	0.69			
	Organizational	107	1.09	0.55			
	No support needed	128	1.13	0.69			
Religious coping	Psychological	94	0.86	0.95	3.34^a^	0.011	0.02
	Emotional	145	1.03	1.04			
	Financial	102	0.63	0.82			
	Organizational	107	0.95	0.93			
	No support needed	128	0.89	1.02			
Seeking emotional support	Psychological	94	1.79	0.88	4.17^a^	0.003	0.03
	Emotional	145	2.04	0.73			
	Financial	102	1.68	0.80			
	Organizational	107	1.91	0.69			
	No support needed	128	1.76	0.84			
Seeking instrumental support	Psychological	94	1.72	0.82	6.20	<0.001	0.04
	Emotional	145	1.92	0.73			
	Financial	102	1.47	0.84			
	Organizational	107	1.73	0.70			
	No support needed	128	1.55	0.84			
Self-distraction	Psychological	94	1.87	0.61	4.29	0.002	0.03
	Emotional	145	1.87	0.62			
	Financial	102	1.69	0.69			
	Organizational	107	1.72	0.67			
	No support needed	128	1.58	0.72			
Denial	Psychological	94	0.90	0.78	5.50^a^	<0.001	0.04
	Emotional	145	0.83	0.68			
	Financial	102	0.72	0.65			
	Organizational	107	0.65	0.68			
	No support needed	128	0.55	0.56			
Venting of emotions	Psychological	94	1.70	0.65	9.32	<0.001	0.06
	Emotional	145	1.78	0.59			
	Financial	102	1.48	0.65			
	Organizational	107	1.56	0.59			
	No support needed	128	1.35	0.69			
Substance use	Psychological	94	0.72	0.88	4.33^a^	0.002	0.03
	Emotional	145	0.51	0.72			
	Financial	102	0.54	0.67			
	Organizational	107	0.43	0.69			
	No support needed	128	0.33	0.58			
Behavioral disengagement	Psychological	94	1.14	0.65	8.55	<0.001	0.06
	Emotional	145	0.94	0.63			
	Financial	102	0.79	0.62			
	Organizational	107	0.79	0.63			
	No support needed	128	0.69	0.60			
Self-blame	Psychological	94	1.60	0.80	10.44	<0.001	0.07
	Emotional	145	1.39	0.80			
	Financial	102	1.00	0.82			
	Organizational	107	1.24	0.87			
	No support needed	128	1.01	0.79			

*—Welch’s correction was applied.

Participants who indicated a need for psychological support and emotional support reported using self-blame more often than did those who indicated a need for financial (*p <* 0.001, *p =* 0.003 respectively). Self-blame is a cognitive judgment related to a belief that not making mistakes is extremely important. However, self-blame causes withdrawal from interpersonal relationships and prevents learning from one’s mistakes. Thus, normal sadness and guilt becomes transformed into depressive disorders [58]. In this case, seeking psychological support seems warranted. People seek to relieve their suffering and solve their problems through utilizing psychological consultations or therapy. However, in contrast to seeking emotional support from significant others within close relationships, individuals seeking psychological support may discount their own agency, responsibility for their decisions, and independent solution-seeking to a greater extent. Additionally, when describing the pandemic situation, it is worth to consider another context of self-blame, namely, the phenomenon of guiltless guilt, that is, guilt without any specific influence on a given situation which is the source of self-blame. This creates a vicious circle which depends psychological suffering [59].

**Fig 7:**
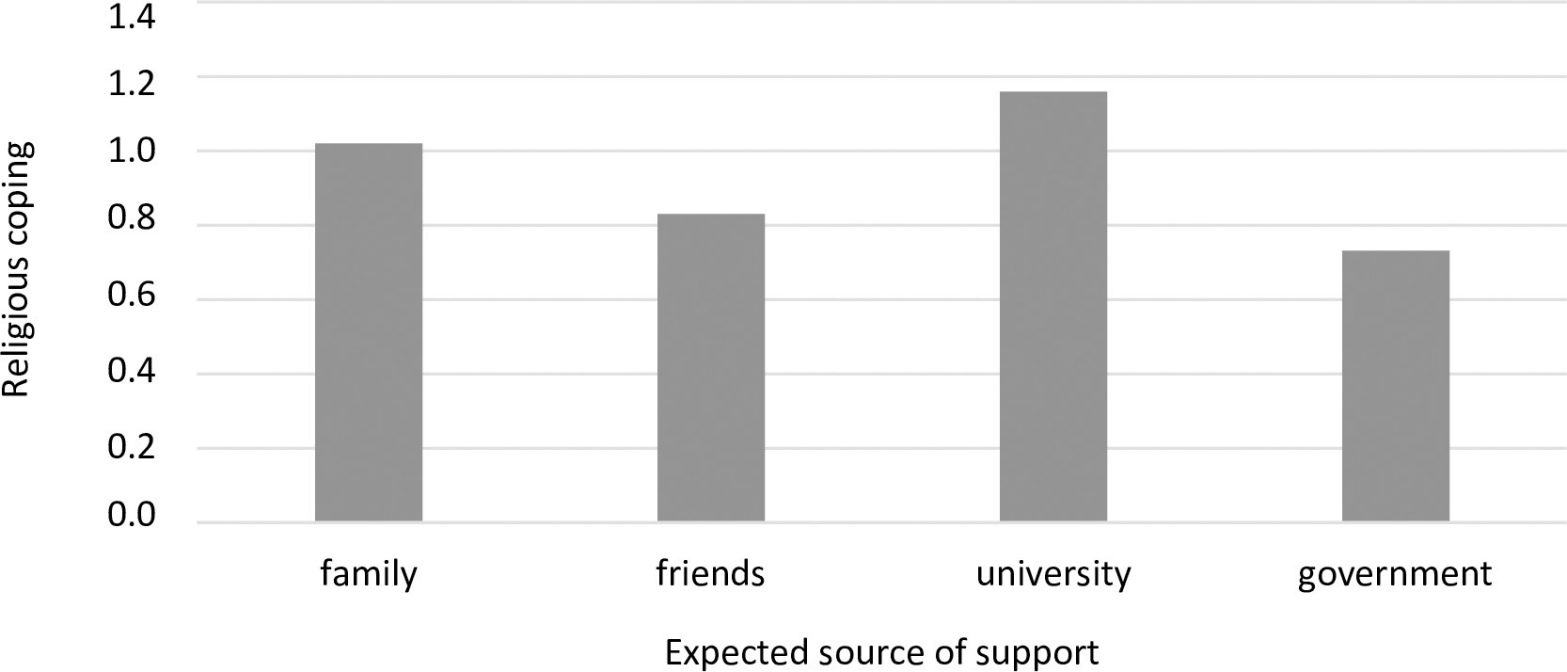
Students’ use of religious coping (mean) and sources of expected support during the COVID-19 pandemic (F = 3.99, p = 0.007, η^2^ = 0.02).

**Fig 8:**
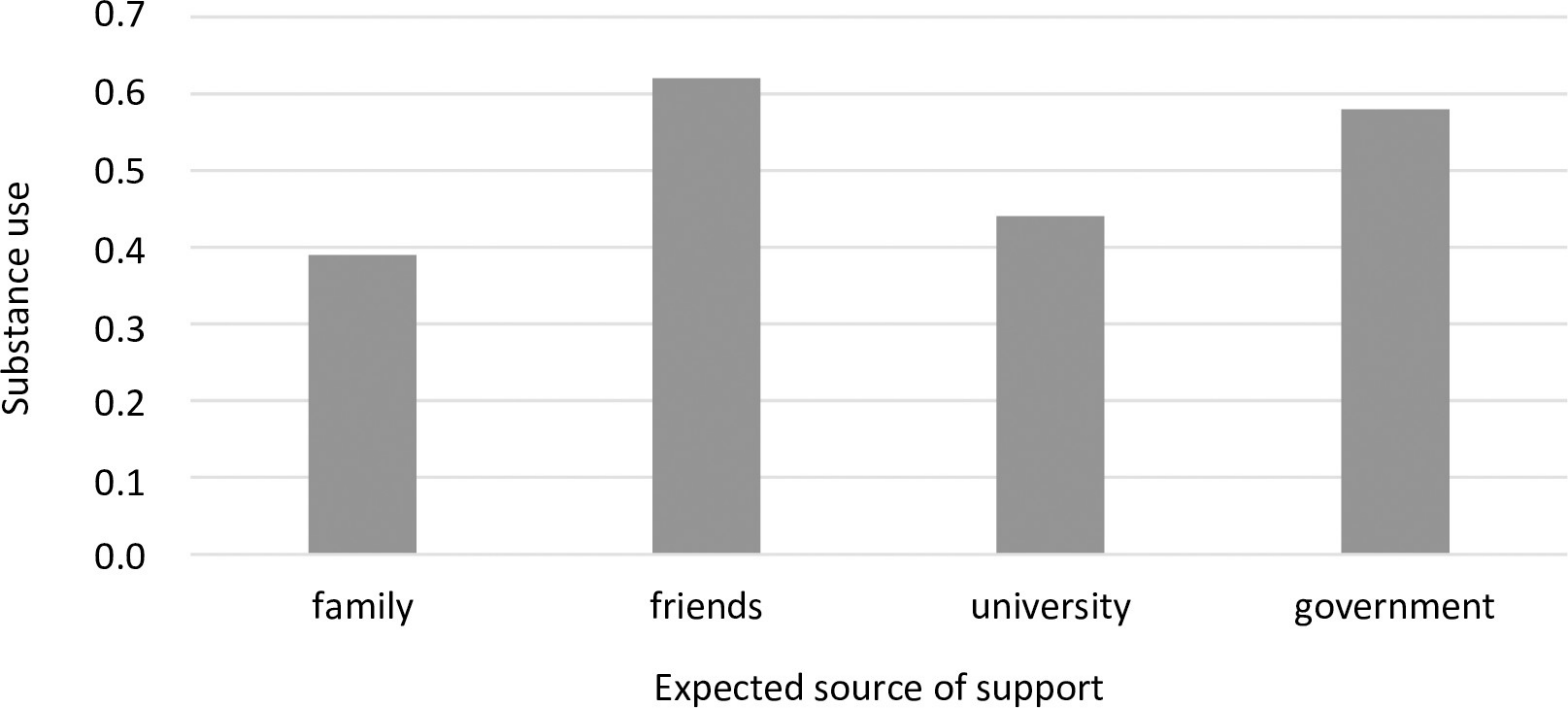
Students’ substance use (mean) and sources of expected support during the COVID-19 pandemic (F = 4.12^a^, p = 0.007, η^2^ = 0.02). * Welch’s correction was applied.

**Fig 9:**
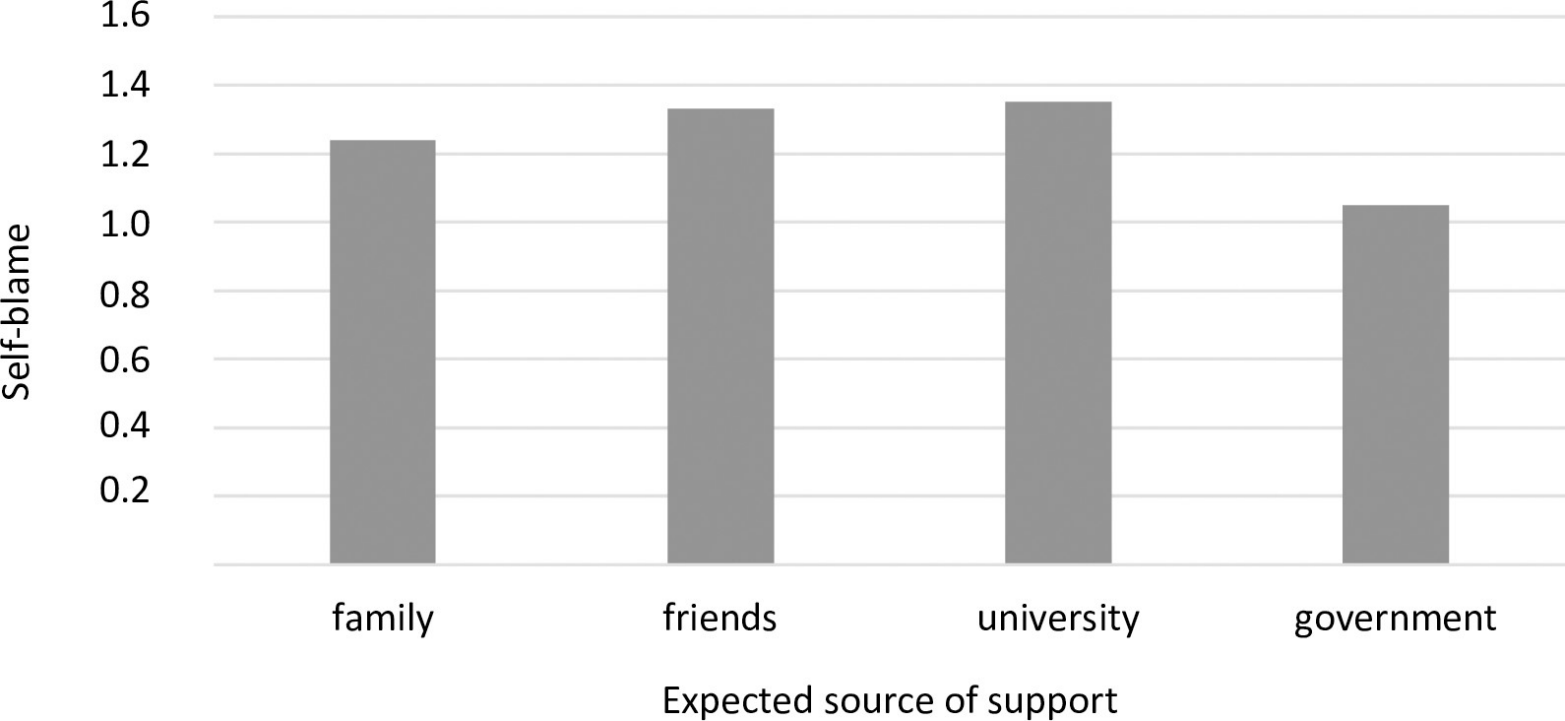
Students’ use of self-blame (mean) and sources of expected support during the COVID-19 pandemic (F = 3.14, p = 0.024, η^2^ = 0.02).

A one-factor ANOVA also revealed significant differences between the groups distinguished by the source of expected support. These differences were significant for three coping strategies: religious coping (Fig 7), substance use (Fig 8), and self-blame (Fig 9). A detailed post hoc analysis revealed that those participants who expected support from the government used religious coping less frequently than those who expected support from their families (*p =* 0.043) or their universities (*p =* 0.026). Participants who expected support from their families reported using the coping strategy of substance abuse significantly less frequently than those who expected support from their friends (*p =* 0.013). In turn, participants who expected support from their friends reported using self-blame significantly more often than did those who expected support from the government (*p =* 0.027).

## Discussion

The aim of the current study was to examine the strategies of coping with stress among Polish university students during the coronavirus pandemic, as well as to assess the type of support they expected. An analysis of the empirical results allows for drawing conclusions on this topic.

The transactional model of stress [33] allowed for identifying a specific relation formed between an individual and their surroundings. The participants in the currents study found themselves in a stressful transaction in which they experienced a real risk in the form of the pandemic situation. Coping is a specific adaptative reaction chosen during the secondary appraisal. It emerges when an individual appraises the situation in terms of harm or risk, that is, as a difficulty. Students differed with respect to their tendency to use specific coping strategies, which does not mean that a given individual’s coping strategies are the same in every situation. Emotion-oriented coping strategies seek to reduce tension and unpleasant emotions which arise in reaction to stressful situations. They are unavoidable particularly when the individual has no influence on the external events. Problem-focused coping involves cognitive and behavioral efforts to reduce stress by trying to solve the problem [33]. Thus, the main condition for appropriate functioning is to develop optimal strategies of coping with stress.

The results of the current study showed that during the stressful situation of the pandemic, which can cause feelings of uncertainty and crisis [60], Polish students mainly chose such coping strategies as acceptance, planning, and seeking emotional support. The current studies showed that students more often use coping strategies which, according to Lazarus and Folkman’s theory [33] are emotion-focused (acceptance, seeking emotional support). On the other hand, planning is a problem-focused coping strategy. These three main strategies seem constructive as they direct people towards a future temporal perspective and might facilitate a reorganization of the values–goals–life plans triad driving the dynamic character of the personality [61], especially in decisive periods of personal crises [62]. It is also related to reframing one’s own life situation. Substance use, denial, behavioral disengagement, and religious coping were used the least frequently by Polish students to cope with stress during the pandemic.

Among all groups of people in education, students exhibited higher levels of emotional problems and pressure related to the changes in the educational and social situation during the pandemic than did primary and high school students [63, 64]. Thus, it was important to assess their need for support and the sources they expect this support from. The results showed that 78% of the students needed support, while 22% did not express such needs. The greatest proportion of students—25%—needed emotional support. This type of support was mainly sought from family (38%) and friends (26%). Participants who expected emotional support simultaneously chose strategies of support seeking and religious coping. Their search for personal resources which would facilitate coping was oriented at close interpersonal relationships. They most likely allowed for conversations which supported another coping strategy preferred by this group, namely, venting and self-blame. Students seeking emotional support–in contrast to those seeking psychological support–likely maintained closer and deeper interpersonal relationships. On the other hand, psychological support was expected by 16% of the participants. They scored the lowest on the strategy of positive reframing. However, they scored higher on substance use, denial, and venting, as well as self-blame. Such a pattern of coping strategies suggests that use of professional psychological help is warranted. These participants showed such difficulties in coping with the pandemic situation that they concluded they should seek professional help from a psychologist.

Those students who expected organizational (18%) and financial (17%) support reported using different coping strategies than did those who expected emotional and psychological support. Such coping strategies as religious coping, support seeking, venting, substance use, behavioral disengagement, and self-blame were lower in this group. Rather, these participants were oriented at gaining concrete material support and support related to organizing their life in the city where they studied, and sometimes also worked to support themselves financially.

Further analyses showed that those students who indicated a need for emotional and social support reported using the coping strategies of positive reframing and acceptance of the pandemic situation less frequently. Need for emotional, organizational, and psychological support was related to typical stressors (studying, pressure to achieve high grades, pass exams, and qualify for scholarships) [65, 66], which remained at similar levels during online teaching. However, it was also related to additional limitations stemming from the digitalization of the teaching process [67]. Moreover, the need for support was also increased by isolation, limitations in social relationships with peers, and limited possibilities for establishing new relationships and realizing affiliative needs [68, 69]. Importantly, these needs concern direct relationships rather than telephone or online contact, as these do not fully mitigate loneliness and do not provide the same amount of support [18]. Seeking real, direct support, the students expressed a perspective of building psychological resilience and improving their emotional state, which corresponds with the results of Bernabé and Botia [70]. Students mainly expressed a need for emotional, organizational, and psychological support from their families and friends in order to maintain a high level of functioning, which might be explained through the perspective of the resilience theory [71, 72]. This is because resilience is strongly related to, among others, perceived emotional support and close, safe relationships with one’s family and friends [73, 74], which create networks of emotional and social support [75, 76]. The ability to use support serves as a buffer for stress and its negative consequences. It can also prevent the deepening of the problems by providing resources for coping when stress occurs. Studies confirm that people with access to support show less reactivity to stress factors and enjoy higher mental health [77].

Next, it was showed that the chosen strategies of coping with stress were related to sociodemographic variables such as gender, age, and place of residence, which was confirmed by Cantor [61]. A detailed analysis revealed gender differences in the use of some specific coping strategies, which is also supported by other studies [78–80]. Women used the strategies of emotional and instrumental support seeking statistically significantly more often than did men. On the other hand, men used humor as a coping strategy more often than did women.

Regarding age, it was shown that younger people who began studying (18-20-year-olds) reported using active coping and planning statistically significantly less frequently than older students (21-24-year-olds, 25-30-year-olds, and those 31 and above). In turn, the higher frequency of using positive reframing and the lowest frequency of using venting of emotions was reported by the oldest students (31 and above). These age-related differences are difficult to relate to previous studies due to methodological differences [81–83]. The strategy of active coping, characteristic for older students, was positively correlated with planning (strong relationship), positive reframing, religious coping, and emotional and instrumental support seeking, which was confirmed by the cluster analysis. Cluster 1 results (statistically significantly higher active coping, planning, positive reframing, humor, religious coping, emotional and instrumental support seeking, self-distraction, and venting, and lower behavioral disengagement) correspond to this profile of active coping.

The youngest students (18–20 years old) did not choose active (adaptive) coping strategies, in contrast to the older students. It is worth noting that, as the youngest persons in the academic community, they have less life and experience and less environmental resources due to the fact that they did not yet develop close and deep social and emotional relationships. This is related to identity development [84, 85]. Additionally, the university is a new setting for such students, which makes it more difficult for them to perceive it as a source of instrumental and organizational support. Thus, the youngest students in particular should be the recipients of complex (psychological, instrumental, possibly also spiritual) support from the university intended to shape appropriate adaptive conditions.

Comparing the full-time and extramural students with respect to their coping strategies, it was found that extramural students scored higher on active coping and positive reframing, and lower on humor, instrumental support seeking, self-distraction, venting, substance use, and self-blame compared to full-time students. It is worth noting that extramural students are usually older than full-time students. Thus, they are at a different developmental period in their lives. They often live with their own families, including their children. They attend classes only during the weekends and are most often employed and financially independent Thus, they have different areas of life activities and exhibit different strategies of coping with stress.

Analyzing the variable of place of residence, it was found that students living in cities with over 100 thousand residents used the coping strategies of planning, humor, and substance use more frequently than did students from smaller towns and villages. These results can be interpreter with reference to Bronfebrenner’s [86] ecological theory. Larger cities have more (both on the mesosystem and the exosystem levels) infrastructural resources, opportunities related to social life, and institutional offers (even during the periods of pandemic-related restrictions). Thus, people living in large cities were subjected to less social isolation during the pandemic than were the people living in rural areas. However, they used religious coping and denial less frequently, which was used more often by students living in villages. Studies on Polish students carried out before the pandemic using the Mini-COPE did not show differences in coping strategies related to place of residence.

The results of the current study allowed for the identification of coping strategies among students. This is important for the process of designing support strategies at universities. Our study also identified the mechanisms of active (adaptive) and passive (maladaptive) coping and directions of support seeking.

Taking into account the current results, future empirical studies can focus on more detailed examinations of the relationships between specific coping strategies used by students. Additional studies on the influence of the later stages of the pandemic on students’ mental health are necessary, as the consequences of this difficult situation may last for a long time, beyond the most intense period of the pandemic.

### Strengths and limitations

The strengths of the current study include an examination of students’ strategies of coping with stress during the pandemic as a global situation which, to some extent, warrants the introduction of monitoring and prevention of the “post-COVID syndrome” in the context of students’ coping with stress and rebuilding social and emotional relationships.

The current results might also serve as a point of reference and comparison for further studies on coping strategies among students in other countries. In turn, this could support the development of local strategies of supporting students in organizing their academic careers and personal lives. This is especially important considering the fact that the occurrence of subsequent pandemics is only a matter of time, as was cautioned by the Director General of the World Health Organization, Tedros Adhanom Ghebreyesus. The UN resolution naming December 27 as the International Day of Epidemic Preparedness acknowledges the disproportional harm they cause in people’s lives and highlights the need for increased awareness, exchange of scientific knowledge, and searching for the best solutions on both the local and national levels. This message finds direct expression in the topic of the current study.

A limitation of the current study is the high proportion of female students of social sciences and humanities in the current sample. In Poland, these programs are more often chosen by women (73%) than men (27%) [87]. This resulted in a high gender imbalance in the current sample.

### Recommendations for universities

The results of the current study lead to formulating several recommendations for universities regarding the organization of teaching in ways that consider the students’ psychosocial functioning to a greater extent. These suggestions include: implementing assessments of students’ psychosocial functioning in order to determine the potential need for emotional, social, and psychological support, and establishing psychological consultation points for students requiring such support.

It also seems warranted to introduce interpersonal training and stress coping workshops for individual student groups. Regarding organizational support, the current results are an argument for providing material support and career counseling in part-time employment for students.

The current results serve as a basis for designing a model of support and-self support solutions for students during the pandemic. The participatory model of intervention development [88] may be particularly useful in this regard. The Participatory Intervention Model (PIM), rooted in participatory action research, provides aa mechanism for integrating theory, research and practice and for promoting involvement of stakeholders in intervention efforts [88]. Based on this model, it seems pertinent to revise the role of the year mentor (*opiekun roku*; in Polish universities, students at each year of their academic program are assigned an academic teacher who meets with the students, acquaints them with the university’s structure and the program, etc.) by including screening assessments of the students’ expected sources of support. Additionally, the role of the university counselor should be created. It is worth noting that the youngest students in particular should be incorporated in the design process for such solutions. This is because the presented study shows that the youngest full-time students showed passive and maladaptive coping strategies. Support solutions designed through the participatory model of intervention should be useful for students, should address their specific needs, and should consider the students’ cultural, organizational, and social contexts, including the context of the pandemic and its consequences. Efforts towards designing adequate interventions may prove insufficient if no attempts are made to understand the students’ beliefs, motivations, practices, language, and culture. Such practices can help universities offer more comprehensive support to students of specific populations. Thus, the particular attention should be draw to the notion of acceptability within PIM, which reflects the perception of the beneficiaries (mainly the university inn this context) as partners in identifying problems and developing the offer of psychological, organizational, and instrumental support solutions created through the process of researching the specificity of the pandemic situation and post-pandemic adaptation. Identifying problems, as well as the scope and range of partnership between the university and its personal and infrastructural resources in planning psychological, organizational, and infrastructural support for students requires an evidence base.

The necessity of carrying out research that would lead to effective practical solutions through PIM is also underscored by Nastasi (et. al.) [89]. The study presented in this article fits this proposal. The current study results showed also that 10% of the sample expecting support from the university. This situation indicates that the current support offer could be insufficient in the context of the pandemic. Thus far, support given to students has been limited mainly to material support–financial support and academic scholarships. Verifying its role as a source of support and an important social environment for its students is also a significant new challenge for universities in the pandemic and post-pandemic reality.

## Conclusions

Studies in this direction should continue in order to examine how students cope with subsequent stages of studying both during the pandemic as well as after its end.

Despite the limitations indicated above, the current results contribute to understanding the social and emotional changes related to the coronavirus pandemic, especially in the area of higher education. Studies on stress and coping among students carried out thus far have not sufficiently considered a range of factors such as the study system (paid vs. free), sources of institutional support (scholarships, student loans, material support), unemployment, or job prospects after graduation. Additionally, similarities and differences in the experience of stress and coping strategies between students in various countries (ethnic and cultural differences) have not been researched to an appropriate degree.

The current study indicates, among others, that younger students who are in the beginning stages of their academic careers cope with stress less effectively. This is largely a consequence of the fact that they do not yet possess appropriate life experience, and thus do not have sufficient competences in coping with difficult situations. This suggests that university administrations should pay particular attention to this group. An obligatory course on coping skills should be recommended for the first year curriculum. This could improve students’ competences, wellbeing, and resilience.

## Supporting information

S1 Data (XLSX)
